# A Small Virus to Deliver Small Antibodies: New Targeted Therapies Based on AAV Delivery of Nanobodies

**DOI:** 10.3390/microorganisms9091956

**Published:** 2021-09-15

**Authors:** Noelia Silva-Pilipich, Cristian Smerdou, Lucía Vanrell

**Affiliations:** 1 Division of Gene Therapy and Regulation of Gene Expression, Cima Universidad de Navarra and Instituto de Investigación Sanitaria de Navarra (IdISNA), 31008 Pamplona, Spain; nsilva.1@alumni.unav.es; 2 Biotechnology Laboratory, Facultad de Ingeniería, Universidad ORT Uruguay, Mercedes 1237, Montevideo 11100, Uruguay; 3 Nanogrow Biotech, CIE BIO Incubator, Mercedes 1237, Montevideo 11100, Uruguay

**Keywords:** adeno-associated virus, AAV, nanobody, antibody, gene therapy

## Abstract

Nanobodies are camelid-derived single-domain antibodies that present some advantages versus conventional antibodies, such as a smaller size, and higher tissue penetrability, stability, and hydrophilicity. Although nanobodies can be delivered as proteins, in vivo expression from adeno-associated viral (AAV) vectors represents an attractive strategy. This is due to the fact that AAV vectors, that can provide long-term expression of recombinant genes, have shown an excellent safety profile, and can accommodate genes for one or several nanobodies. In fact, several studies showed that AAV vectors can provide sustained nanobody expression both locally or systemically in preclinical models of human diseases. Some of the pathologies addressed with this technology include cancer, neurological, cardiovascular, infectious, and genetic diseases. Depending on the indication, AAV-delivered nanobodies can be expressed extracellularly or inside cells. Intracellular nanobodies or “intrabodies” carry out their function by interacting with cell proteins involved in disease and have also been designed to help elucidate cellular mechanisms by interfering with normal cell processes. Finally, nanobodies can also be used to retarget AAV vectors, when tethered to viral capsid proteins. This review covers applications in which AAV vectors have been used to deliver nanobodies, with a focus on their therapeutic use.

## 1. Adeno-Associated Viral Vectors

Adeno-associated virus (AAV) is a small, non-enveloped, and non-pathogenic virus that belongs to the Parvoviridae family. AAV cannot replicate by itself, needing the presence of a helper virus, such as adenovirus, for propagation. The AAV particle contains an icosahedral capsid consisting of 60 copies of three related proteins, VP1, VP2, and VP3 (with a 1:1:8 ratio), which surrounds a 4.7 kb single-stranded DNA genome flanked by two palindromic inverted terminal repeats (ITRs) of 145 bp. The viral genome comprises two open reading frames: *rep* and *cap*. *Rep* encodes four non-structural proteins, while *cap* encodes the capsid proteins and the assembly-activating protein (AAP) [1].

In AAV vectors, all viral genes are replaced by the gene of interest [1], leaving only the ITRs, which serve as origin of replication and packaging signals during vector production. These sequences also allow long-term episomal persistence of the therapeutic transgene in the nucleus of transduced cells [2].

For AAV vector production, *rep* and *cap* genes are provided in *trans*, along with the necessary helper virus genes [3]. There are at least 12 serotypes of AAV with different tropisms, and more than 100 AAV variants, from which a wide variety of mutants have been generated to optimize the delivery of genes to different target tissues [4]. To take advantage of the full potential of such variants, it is possible to package the therapeutic construct flanked by AAV2 ITRs into capsids of different serotypes (or mutants), a process referred to as pseudo-packaging [5].

AAV vectors are highly stable and easy to produce. Furthermore, they have an excellent safety profile and have shown high transduction efficiency in various target tissues, transducing both quiescent and dividing cells, and allowing long-term expression of the transgene in animal models [6]. For these reasons, they have become the leading viral vectors for in vivo gene delivery, not only in pre-clinical investigation but also to treat a variety of human diseases in clinical stages, including bleeding, musculoskeletal and lysosomal storage disorders, inherited blindness, and neurodegenerative diseases, among others [4,7,8].

As a limitation, the packaging capacity of AAV is low, being approximately 5 kb. Furthermore, the recombinant episomal forms of AAV do not replicate during mitosis of dividing cells, so the therapeutic effect would fade after several generations [9]. Nevertheless, strategies to maintain AAV episomes after cell division by incorporating autonomous replication units into the vector genome [10] or to integrate rAAV genomes at specific sites have been developed [11,12].

Moreover, the host’s immune response can be a major obstacle in AAV vector translation to the clinic due to both innate [13] and adaptive immune responses that can hamper AAV transduction (the latter consisting of cytotoxic T lymphocytes and antibodies against capsid proteins and/or the therapeutic protein) [14]. To counteract the immune-mediated clearance of transduced cells, transient immunosuppressive treatments, AAV capsid engineering strategies, or a combination approach have been proposed (reviewed in [4]).

While AAV vectors have gained significant attention as gene therapy vectors for rare inherited monogenic diseases, their use is not limited to these pathologies. Thanks to a number of factors including early approval by regulatory agencies, an excellent safety profile, availability of large-scale production, and the versatility of vector tailoring through genetic engineering, AAV vectors are showing a great potential to treat a broad range of diseases. These vectors are particularly suitable to provide sustained expression of therapeutic proteins in serum, including therapeutic antibodies. In fact, studies in non-human primates have shown that AAV vectors can provide long-term expression of broadly neutralizing antibodies against human immunodeficiency virus (HIV) when delivered systemically [15]. This has led to a recent clinical trial in which this approach has shown a good safety profile [16].

## 2. Therapeutic Antibodies

### 2.1. Conventional and Heavy-Chain Antibodies

The field of personalized therapy using monoclonal antibodies (mAbs) has been in continuous expansion for more than three decades, when the first therapeutic antibody was approved for clinical use [17]. The antibody market has become dominant in the biopharmaceutical industry since 2009 [18], with numerous mAbs marketed in the US and the EU for the treatment of a wide array of diseases including cancer, autoimmune diseases and infections [19,20]. Despite the great success achieved by mAbs, several limitations, such as poor tissue penetration, immunogenicity, and high production costs, suggest that there is still room for improvement.

**Figure 1:**
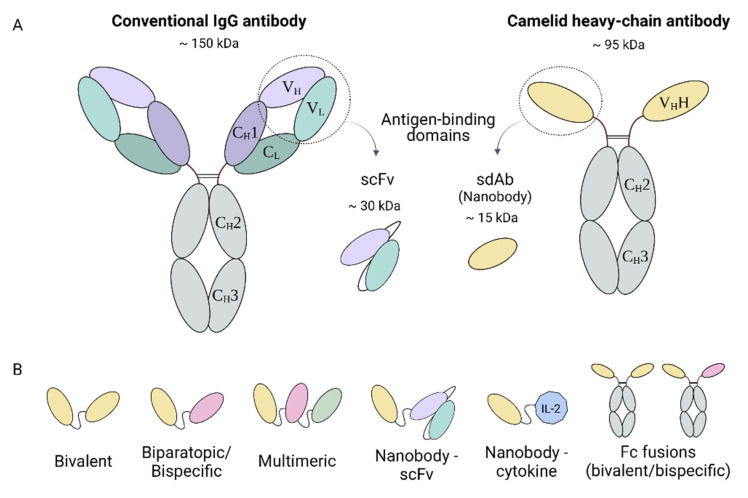
Graphical representation of conventional and heavy-chain antibodies. (**A**) Schematic structure of a conventional IgG antibody, composed by two heavy and two light chains, and a camelid heavy-chain antibody, consisting of two heavy chains. (**B**) Nanobody-based engineered molecules. scFv, single-chain fragment variable.

Conventional antibodies are complex molecules composed of two identical heavy chains and two identical light chains connected by interchain disulfide bonds and non-covalent interactions, forming a Y-shaped heterotetramer (Figure 1A). They are large molecules, with a molecular size of 150 kDa. The C-terminal halves of the heavy chains comprise the crystallizable fragment (Fc), which grants the antibody with secondary functions such as antibody-dependent cellular cytotoxicity (ADCC) or complement-dependent cytotoxicity (CDC). The light chain and the two N-terminal domains of the heavy chain comprise the antigen-binding fragment (Fab). Antigen recognition is provided by the variable domains of the heavy (V_H_) and light (V_L_) chains, specifically by three hypervariable loops in each domain that comprise the complementary-determining regions (CDRs 1, 2, and 3), flanked by four more conserved regions known as framework regions (FRs 1, 2, 3, and 4) [21].

The large and complex structure of conventional antibodies leads to several drawbacks that have prevented the widespread use of these molecules. Due to their complexity and posttranslational modifications, therapeutic mAbs are generally expressed in mammalian cells, leading to high production costs [22]. mAbs have a limited physicochemical stability [23], but once administered, they have a long serum half-life that may cause toxicities related to the treatment [22]. Although the technology for generation of fully humanized antibodies has improved their immunogenic profile, protein aggregation is still a concern because it can increase their immunogenicity while decreasing activity [24,25]. In addition, the large size of mAbs can obstruct their homogeneous distribution in tissues, especially in solid tumors where penetration tends to be inefficient [26], and in the central nervous system, due to their inability to cross the blood–brain barrier [27].

Naturally occurring heavy-chain antibodies (HCAbs) were found in camelids (dromedaries, camels, llamas, alpacas, guanacos, and vicuñas) nearly 30 years ago [28]. These antibodies are homodimers consisting solely of two identical heavy chains, lacking both the light chain and the constant domain 1 (C_H_1) of the heavy chain (Figure 1A). As a result, they are smaller than conventional antibodies, having a molecular mass of approximately 95 kDa. The variable antigen-binding domain of HCAbs (called V_H_H) retains full antigen-binding potential despite lacking the light chain. With dimensions of 4 nm × 2.5 nm × 3 nm, the V_H_H domain is considered to be the smallest naturally occurring antigen-binding fragment to date and has inspired the company Ablynx to coin the term “nanobody” [29,30].

### 2.2. Nanobodies

Structurally, nanobodies are similar to the V_H_ domain of conventional antibodies, with four FRs and three CDRs. The homology between nanobodies and the V_H_ domain of human immunoglobulins of family III is above 80% [31]. Despite this high level of homology, there are some key differences between V_H_ and V_H_H domains. First, there is an increased frequency of polar amino acids in the FR2 of nanobodies compared to human antibodies. As a result, nanobodies present higher hydrophilicity, leading to an increased solubility in polar solvents and enhanced physicochemical stability compared to the variable fragment of conventional antibodies [32]. Second, the CDR3 tends to be longer in nanobodies than in conventional antibodies, having on average of 18 amino acids versus 14 amino acids in human antibodies. In nanobodies, the CDR3 is the main region implicated in antigen binding, assisted by CDR2 and CDR3, and some FR residues. This extended CDR3 loop is able to form finger-like structures or convex paratopes, allowing the binding to small cavities or concave epitopes (mainly conformational epitopes), such as catalytic sites of enzymes. In contrast, conventional antibodies are better at recognizing small chemical groups (haptens), peptides, or flat epitopes on proteins. These differences in antigen preference suggest non-overlapping functions for both types of antibodies [33,34].

The unique features of nanobodies make them attractive biotechnological tools for many applications. Their single-domain nature and small size simplify their genetic manipulation, and different relatively simple strategies can be used to generate specific nanobodies [35]. V_H_H libraries can be generated from naïve [36,37] or immunized animals [38], with full retention of functional diversity as no repairing of V_L_ and V_H_ domains is required, in contrast to conventional antibody libraries. Synthetic libraries can also be used, replacing animal experimentation [39,40]. Selection of target-specific nanobodies from these libraries can be performed through different techniques, such as phage or yeast display [35], and subsequent in vitro affinity maturation might improve binding properties [41]. Furthermore, genetic manipulation after the selection of nanobodies is facilitated due to their single-domain nature, making it possible to design multivalent formats of nanobodies or fusing them to other proteins (Figure 1B) [42].

Nanobodies have been successfully expressed in different systems obtaining high yields, including bacteria, yeast, plant cells, insect cells, and mammalian cells, as revised elsewhere [43]. This is possible thanks to their high solubility and physicochemical stability, single-domain nature, and the fact that posttranslational modifications are not needed. The possibility of using inexpensive systems such as *E. coli* for nanobody production makes them very attractive tools for many applications.

### 2.3. The Therapeutic Potential of Nanobodies

Although the development of nanobodies for research and diagnostic purposes has experienced exponential growth in the last decade, advances in nanobody-based therapies have been more modest. The small size of nanobodies can be a double-edged sword for therapy: on the one hand, they show a homogenous distribution and a high and fast tissue penetration when delivered in vivo, but on the other hand, their serum half-life is very short, as their size is below the renal filtration cutoff of ~60 kDa [44]. While this characteristic is interesting for certain diagnostic applications such as molecular imaging [45], where fast blood clearance is desirable, it represents an obstacle for long-term therapeutic purposes. Several strategies can be employed to improve the pharmacokinetics of nanobodies, including fusion to an albumin-binding nanobody [46], or to an Fc domain [47].

Currently, numerous nanobody-based therapeutics are under clinical trials for the treatment of a variety of diseases, including cancer, autoimmune diseases, and viral infections [34], which are summarized in Table 1. One of the main concerns of using antibodies in humans is their potential immunogenicity, especially upon repeated administrations. Immunogenicity of nanobodies is normally low due to the high level of homology between nanobodies and human V_H_ domain [48]. In addition, humanization of nanobodies can be performed to reduce potential adverse effects [49]. A phase I clinical trial using a non-humanized nanobody showed no adverse effects, although it was administered only once for imaging purposes [50]. In another study, development of hepatotoxicity led to the early termination of phase I clinical evaluation of TAS266, an agonist humanized tetravalent nanobody targeting Death Receptor 5. Preexisting antibodies against TAS266 were found in those patients that developed adverse effects [51]. Although autoantibodies against framework regions of human V_H_ domains can be found in a proportion of healthy individuals [52], TAS266 immunogenicity may be more related to its artificial tetrameric nature and strong biological activity [51], as no adverse events have been described for other bivalent or trivalent nanobodies [48]. Recently, a platform for the generation of fully human heavy-chain antibodies in genetically engineered rats has been described [53]. This kind of strategy could offer in the future a straightforward procedure for the generation of human nanobodies with an improved safety profile for clinical use.

Recently, the first nanobody-based therapeutic (caplacizumab) was approved by the Food and Drug Administration and the European Medicines Agency for treatment of acquired thrombotic thrombocytopenic purpura (aTTP) [63]. aTTP is a blood disorder in which the activity of von Willebrand factor (vWf)-cleaving protease ADAMTS-13 is highly inhibited, which leads to the formation of vWf-platelet aggregates and microvascular thrombosis. Caplacizumab is a bivalent nanobody with a molecular mass of 28 kDa that binds to vWf and inhibits platelet aggregation. Subcutaneous administration of caplacizumab has shown to reduce disease recurrence and aTTP-related deaths, compared to patients receiving placebo [54]. This groundbreaking approval could give a boost to the development of new nanobody-based therapies in the years to come.

### 2.4. Nanobodies and Gene Therapy

Delivery of therapeutic antibodies using gene therapy vectors has been in continuous progress for more than two decades [64], when the first attempt was reported using an adenoviral vector [65]. Viral vectors are the most used delivery vehicle in pre-clinical and clinical trials due to their remarkable gene delivery efficiency [66]. Several strategies to enhance the efficacy and safety of this type of therapies have been described, including regulation of expression using inducible promoters [67], and promoting tissue-specific gene transfer and expression, which can be obtained by selecting the appropriate vector, the route of administration, and tissue-specific promoters. Local expression would not only increase the bioavailability of the therapeutic antibody in the target organ, but also reduce its dissemination to the blood, preventing potential toxicities. Although AAV vectors have been widely used to express mAbs in vivo, expression of nanobodies with these vectors has only been recently explored.

Nanobodies are particularly suitable agents for gene therapy due to their small size and single-gene nature, which facilitates the design of multivalent constructs and nanobody-protein fusions (Figure 1B). Interestingly, nanobodies can be expressed both as extracellular and intracellular proteins, broadening their potential applications. Intracellular antibodies, known as intrabodies, can be used to treat diseases caused by intracellular processes. Nanobodies have the advantage over mAb, single-chain fragment variable (scFv) and Fabs, of being highly stable even in the reducing environment of the cytoplasm, where they can fold properly in the absence of disulfide bonds [68,69]. Nevertheless, as this is not a general statement for all nanobodies, optimizing library design, selection methods, and sequence optimization is essential for attaining functional intrabodies [70,71].

**Figure 2:**
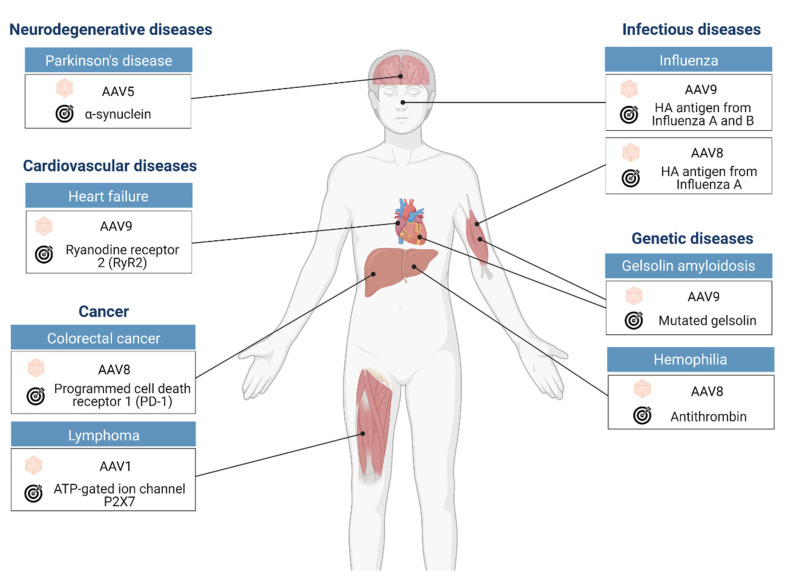
Therapeutic applications of AAV-mediated nanobody gene delivery in preclinical studies discussed in this review. For each study, this figure illustrates the disease model, the AAV serotype used, the target organ, and the target of the therapeutic nanobodies.

This review focuses on the current preclinical achievements in nanobody gene therapy using AAV vectors for the treatment of various diseases including cancer, genetic, infectious, and cardiovascular diseases (Table 2 and Figure 2).

## 3. AAV Vectors Expressing Nanobodies for Treatment of Genetic Disorders

The use of gene therapy to treat genetic disorders is usually based on the delivery of the correct gene to target cells through an appropriate vector. However, some genetic diseases may also benefit from the use of therapeutic antibodies that target proteins or altered protein modifications that contribute to the pathogenesis of the disease. In this scenario, the use of antibody gene therapy is of particular interest, since it would be more cost-effective than repeated administration of antibody proteins. AAV vectorsexpressing therapeutic nanobodies have been used in experimental models of genetic diseases that include gelsolin amyloidosis (AGel) and hemophilia A and B.

AGel is an autosomal dominantly inherited disease that is produced by a point mutation in the gelsolin gene. This mutation results in sequential cleavage of gelsolin by furin and membrane type 1-matrix metalloproteinase (MT1-MMP) during its secretion, leading to the production of a small 8-kDa peptide that aggregates, forming cross-beta-sheet amyloid fibrils and plaques. AGel patients usually experience neurological, ophthalmological, and dermatological symptoms, with no specific treatments available. The group of Jan Gettemans (Ghent University, Belgium) had previously developed a llama nanobody against gelsolin (GSN Nb11) able to function as a molecular chaperone, blocking the cleavage of this protein by furin [87]. They showed the functionality of this approach by crossbreeding AGel mice with transgenic mice able to express GSN Nb11, which resulted in improved muscle contractility. They have recently evaluated the possibility of delivering GSN Nb11 from AAV vectors, something that could facilitate the use of this approach in the clinic [72]. For this purpose, they combined GSN Nb11 with a second nanobody able to inhibit MT1-MMP using a single bispecific format, with the aim to inhibit both proteolytic cleavages simultaneously. Interestingly, the two nanobodies were separated by an MT1-MMP-sensitive peptide sequence which also functioned as an MT1-MMP protease decoy. An AAV9 vector administered to neonate AGel mice was able to successfully express this bispecific nanobody, which could be detected in serum at constant levels up to three months of age. Most importantly, AGel mice treated with the AAV9 expressing the bispecific nanobody showed a reduction in gelsolin amyloid burden, which translated into improved muscle contractile properties. In fact, in this study, the decline in the speed of muscle contraction during fatigue in treated AGel mice was very similar to the one observed for wild-type mice.

Hemophilia is a genetic disease in which a coagulation factor deficiency leads to spontaneous bleeding, as well as bleeding after injuries or surgery. Gene therapy for hemophilia A and B using AAV vectors expressing factors VIII and IX, respectively, has shown very promising results in clinical trials [88]. A different approach to avoid bleeding in these patients is the use of antithrombin inhibitors, since this protein blocks thrombin, the enzyme responsible to convert fibrinogen into fibrin. Antithrombin blockade can be achieved by llama-derived nanobodies, as shown recently by Barbon et al. [75]. As observed with other nanobodies mentioned in this review, their potency was very limited when used as monomers. However, by combining two different nanobodies with a flexible linker, it was possible to obtain a potent inhibitor of antithrombin activity even in the presence of heparin. This bispecific nanobody was able to restore hemostatic balance in hemophilia A mice when given as a recombinant protein. Most interestingly, similar results were obtained when the anti-antithrombin bispecific nanobody was produced from the liver in hemophilia A and B mice that received an AAV8 vector able to express it. The fact that AAV therapy in these mice was long-lasting and showed no toxicity suggests that this type of novel approach could be useful for treating different types of genetic clotting disorders.

## 4. AAV Vectors Expressing Nanobodies to Treat Heart Failure

An interesting approach to treat heart failure is based on the use of agents able to restore normal calcium circulation in cardiomyocytes, which is commonly altered in patients suffering a heart attack. This is usually due to an impaired function of ion channel proteins such as RyR2, sarco/endoplasmic reticulum Ca^2+^ ATPase, and the Na^+^-Ca^2+^ exchanger. In the case of RyR2, hyperphosphorylation has been postulated as an important pathologic mechanism for myocardial injury and heart failure development, representing an interesting pharmacological target. Following this rationale, Li et al. developed a camel nanobody that could inhibit RyR2 phosphorylation [73]. In order to deliver this nanobody in vivo, they used an AAV9 vector and tested its functionality in a rat model of ischemic heart failure induced by coronary artery ligation. Nine weeks after injury, they observed that animals treated with AAV particles expressing the anti-RyR2 nanobody showed a significant improvement compared to untreated controls, indicated by a lower amount of cardiac fibrosis and a higher heart weight/body weight. These effects seemed to be mediated by a reduction in calcium leakage from cardiomyocytes in rats, improving their contractility. In this particular approach, the advantage of AAV delivery is based on the fact that the anti-RyR2 nanobody can only work intracellularly since the target protein is not secreted. This type of “intrabodies” can greatly benefit from the use of AAV vectors that can mediate their expression in target cells.

## 5. AAV Vectors Expressing Nanobodies for Treatment of Neurodegenerative Diseases

Neurodegenerative diseases are a group of heterogeneous illnesses including Alzheimer’s disease, Parkinson’s disease (PD), frontotemporal dementia, and Huntington’s disease, among others. Despite their clinical and epidemiological heterogeneity, most of them are characterized by precipitation of misfolded endogenous proteins, which leads to progressive neuronal dysfunction and death [89,90]. Different observations suggest that protein aggregates can spread from cell to cell, contributing to the pathology of these diseases [91]. Thus, many therapeutic strategies are aimed at preventing this phenomenon by using passive or active immunization. Protein aggregation inside cells can also be inhibited by targeting the particular mRNA or protein responsible of the disease, alleviating neuronal toxicity [92,93]. AAVs are very attractive vectors for the delivery of therapeutic genes to the central nervous system due to their good safety profile, the neuronal tropism of some serotypes, and their long-term gene expression.

PD is the most common type of synucleinopathy, a group of diseases characterized by misfolding and aggregation of α-synuclein (α-syn). Therapeutic strategies for PD under investigation include the use of intrabodies against α-syn [94]. In particular, one nanobody (NbSyn87) and one scFv isolated from a non-immune human antibody library (VH14) [95] have shown protective activity in vitro [96]. Fusion of these intrabodies to a highly charged proteasomal-targeting signal (PEST) increased their solubility and enhanced the degradation of α-syn [97]. The therapeutic effect of the intrabodies has been evaluated in a PD rat model, in which overexpression of human α-syn in the substancia nigra (SN) was achieved using an AAV5 vector. Three weeks post-lesion, animals received a single injection of a second AAV5 vector expressing VH14 or NbSyn87 fused to PEST into the SN. Both intrabodies showed alleviation of pathogenic α-syn aggregation and improved motor functions; however, VH14 was superior at preserving overall nigrostriatal health. This could be attributed to the fact that NbSyn87 seemed to induce some degree of neuroinflammation [74]. These studies showed that intrabodies can be delivered to the central nervous system using AAV vectors and that they can have a beneficial effect on reducing intracellular protein accumulation. The development of combinatorial strategies that target protein accumulation both in intracellular and extracellular compartments hold great promise for the future.

## 6. AAV Vectors Expressing Nanobodies to Fight Viral Infections

Vaccines remain the main method of infectious disease control. However, their poor immunogenicity in certain high-risk patient groups, as well as the time needed to generate a potent immune response, are some of their shortcomings. In these cases, passive immunization using antibodies may be a feasible strategy to confer a quick protection during epidemics or pandemics [98]. Due to their unique properties, nanobodies have great potential as prophylactic and therapeutic agents to treat infectious diseases caused by a variety of pathogenssuch as viruses [99]. Several virus-neutralizing nanobodies have been described, including nanobodies against HIV [100], Hepatitis B virus [101], Middle East respiratory syndrome coronaviruses (MERS-CoV) [102], human respiratory syncytial virus (RSV) [103,104], rotavirus [105,106], influenza virus [103,107], and, more recently, SARS-CoV-2 [108,109,110].

Influenza virus represents a major public health concern, causing yearly epidemics and occasional pandemics. Due to the antigenic drift of the virus, vaccines have to be reformulated every year based on the predicted circulating virus strains for the upcoming season. Current vaccines usually induce strain-specific immunity, and they can be ineffective if there is a mismatch between the circulating viruses and vaccine strains [111]. In addition, these vaccines usually do not confer protection against emergent pandemic strains, such as the H1N1 in 2009, for which production of a strain-specific vaccine became necessary [112]. Limitations of seasonal influenza vaccines and the constant threat of a new influenza pandemic urge for the development of a wider range of strategies to tackle these viruses.

Broadly neutralizing antibodies (bnAbs) that recognize conserved influenza virus epitopes, especially within the hemagglutinin (HA) stem domain, have been isolated from humans, suggesting that a universal immunity against influenza virus may be possible [113]. Several conventional bnAbs against this protein are currently under clinical evaluation [114,115]. In terms of prophylaxis, this strategy would probably be too expensive and impractical to be widely implemented, because it would require 1) the formulation of a cocktail of bnAbs to achieve full coverage against both influenza A and B viruses, and 2) multiple administrations of high doses of bnAbs throughout the influenza season. A potential simpler strategy would be to deliver bnAbs using gene therapy. In this regard, AAV vectors encoding conventional bnAbs have been tested in preclinical studies with promising results [116,117,118]. However, the bnAbs described to datelack of sufficient influenza A and B cross-reactivity, which means that a combination of at least two different bnAbs should be used to achieve full protection [119]. This could represent a problem when using AAV vectors due to its limited packaging capacity (<5 kb).

Interestingly, elucidation of the structure of two bnAbs in complex to HA has shown that, in both cases, the light chain is unnecessary for antigen binding [120,121]. Following this observation, different groups have attempted to isolate broadly neutralizing nanobodies against influenza. Hufton and colleagues [122] identified a nanobody (R1a-B6) with broad cross-subtype neutralizing activity in vitro. A bivalent format of this nanobody increased the breadth of cross-neutralization to more divergent subtypes, suggesting that the weak affinity interactions of monovalent R1a-B6 could be improved by dimerization [122]. The prophylactic potential of R1a-B6 was evaluated in a mouse model using AAV8 to express this nanobody as a monovalent molecule or fused to mouse IgG1 or IgG2a Fc domains [76]. A single dose of AAV8 given intramuscularly provided robust expression of R1a-B6-Fc fusions in sera, that was sustained for at least 6 months [76]. Mice in this study were challenged with lethal doses of two different pandemic strains H1N1 (A/California/07/2009) and H5N1 (A/Vietnam/1194/2004) at day 42 post-AAV administration. While mice in control groups succumbed to the infection quickly, mice expressing R1a-B6 fused to IgG1-Fc or IgG2a-Fc showed no symptoms of infection [76]. Mice treated with monovalent R1a-B6 showed a delay in the onset of symptoms but eventually succumbed to the infection, underlying the importance of Fc fusions [76]. It would be interesting to evaluate if Fc-mediated dimerization of nanobodies is capable of enhancing the breadth of cross-neutralization activity in vivo for other subtypes, as it was shown in vitro [122].

In another study, Laursen and colleagues isolated four broadly neutralizing nanobodies that were capable of neutralizing viruses from different groups of influenza A and B [77]. To increase their potency and breadth, the four nanobodies were fused using peptide linkers to generate a multiple-domain antibody (MDAb), which was additionally fused to Fc domains that conferred effector functions (human IgG1 or mouse IgG2a). This construct, named MD3606, showed great potential in prophylactic experiments when administered intravenously, outperforming bnAb CR9114 [113], which has been widely used in influenza virus research. MD3606 was also delivered using an intranasally administered AAV9, and, seven days later, mice were challenged with three different influenza viruses: H1N1 (A/Puerto Rico/8/34-MA), H3N2 (A/Hong Kong/1/68-MA), and B virus (B/Lee/40-MA). In this study, rAAV9-delivered MD306 provided complete protection in all cases, and abrogation of the Fc secondary functions substantially decreased the protective potential of MD3606 [77].

An interesting route to deliver AAV vectors expressing nanobodies against respiratory pathogens is the nasopharyngeal administration. By using this route, AAV9 vectors have been able to express antibodies up to four months in rhesus macaques [117]. Although the expression is not sustained, the rapid onset of protection observed in mice (seven days after AAV administration) and the breadth of neutralization makes this an appealing strategy in pandemic scenarios, where vaccines are not immediately available. In addition, AAV9 can be re-administered in the airways without loss of efficiency [123,124], which could extend the time of protection. Moreover, combining intranasal and intramuscular AAV administration could provide the double benefit of local and prompt protection from nasal expression, and long-term systemic protection from transduced muscle cells [76,77]. Importantly, these routes of administration have been shown to circumvent pre-existing neutralizing antibodies to AAV capsids in mice [123] and rhesus macaques [125].

## 7. AAV Vectors Expressing Nanobodies for Cancer Treatment

The discovery of immune system checkpoints has been a major breakthrough for cancer therapy. Although immune checkpoints constitute a natural mechanism to avoid toxicity and autoimmunity, many tumors express these molecules to escape immune attack [126]. One of the immune checkpoints that many tumors exploit to evade the immune response is the programmed death-1 (PD-1) / programmed death ligand 1 (PD-L1) axis, whose blocking by monoclonal antibodies (mAbs) has shown clinical efficacy in the treatment of many types of cancer [127]. However, this therapeutic strategy requires the repetitive systemic administration of high doses of mAbs that often leads to adverse effects, such as autoimmune events [128]. In addition, due to their large size (~150 kDa), mAbs show limited tumor penetration, which decreases their performance. To overcome these limitations, nanobodies represent very attractive tools because their small size allows high extravasation and tissue penetration [30]. However, as mentioned earlier, this can also be a downside, as they are rapidly eliminated from the bloodstream through renal clearance. One strategy to counteract this drawback could be the use of gene therapy vectors, such as AAV, that can continuously express nanobodies in vivo at therapeutic levels.

In fact, this approach was used by us to deliver in vivo a novel anti-PD-1 nanobody (Nb11) capable of blocking the PD-1 / PD-L1 interaction for mouse and human molecules [78]. This study showed that sustained AAV-mediated expression of the Nb11 prevented colon adenocarcinoma tumor formation in 30% of mice, significantly increasing survival without evidence of toxicity or autoimmune events. These data suggest that continuous expression of immunomodulatory nanobodies from long-term expression vectors could have antitumor effects with low toxicity.

In a different approach, Demeules et al. have expressed in vivo two nanobodies that can modulate the function of mouse P2X7, an ATP-gated ion channel with a crucial and complex role in cancer that is not completely understood [79]. Although there is evidence that P2X7 is associated with proinflammatory activity that could be pro-tumorigenic [129], it is also linked to the promotion of an adaptive antitumor immune response [130]. In order to elucidate the role of P2X7 in cancer, the authors generated AAV1 vectors encoding dimers of either antagonist or agonist nanobodies against P2X7 [79]. They were able to show that constant in situ expression of the P2X7 antagonistic bivalent nanobody induced a complete tumor regression in 40% of mice, significantly improving survival in a mouse lymphoma model. In contrast, AAV-mediated expression of the agonistic nanobody dimer did not significantly influence tumor growth in this model. By using nanobodies with different activities, this study demonstrated that P2X7 could be used as a target for anti-tumor treatment.

## 8. Other Applications of AAV Nanobody Delivery

Thanks to the beneficial properties of nanobodies as small entities with high stability and specificity, they are highly valued as versatile tools to study and manipulate proteins within living cells (Table 2). This unique property of nanobodies has led to the development of additional research applications that have also benefited from the use of AAV vectors for delivery, as it will be discussed in this section.

A simple example in which AAV delivery of nanobodies can be used to study biological functions is represented by a situation in which constant release of a nanobody is achieved in a particular type of cell, as in the case of the study presented above by Demeules et al. [79], which aimed to elucidate the function of P2X7 in the tumor context. Following a similar rationale, Zhang et al. described an agonist nanobody for the Hedgehog pathway, which is involved in embryonic tissue patterning and postembryonic regulation of tissue homeostasis and regeneration [80]. By specifically targeting conformational forms of Hedgehog receptor Patched1 (PTCH1), the nanobody stabilized this protein, disrupting its conformational cycle and, thus, its transport activity, leading to pathway activation. Although selective delivery of this nanobody by AAV vectors could increase tissue regeneration around transduced cells, avoiding systemic effects such us overgrowth of mesenchyme and fibrosis in multiple organs [131], this was not addressed in this study. Instead, the authors used systemic delivery to demonstrate that prolonged and sustained expression of anti-PTCH1 nanobody activated the Hedgehog signaling pathway in lingual epithelium and skin. This approach can help to deepen the mechanistic aspects of this pathway and evaluate possible therapeutic applications.

A milestone in the use of nanobodies in research was the development of chromobodies, which consist of fusion proteins between an intracellular nanobody and a fluorescent protein. Chromobodies can be expressed from AAV vectors and are useful to visualize cellular structures and processes without interfering with the target’s function [132]. An example of this application is the chromobody formed by an anti-actin nanobody and the far-red fluorescent protein mNeptune2. The intracranial delivery of this chromobody with a neuron-specific AAV1/2 hybrid vector allowed to super resolve actin filaments and study dendritic spines dynamics in mice [81].

**Figure 3:**
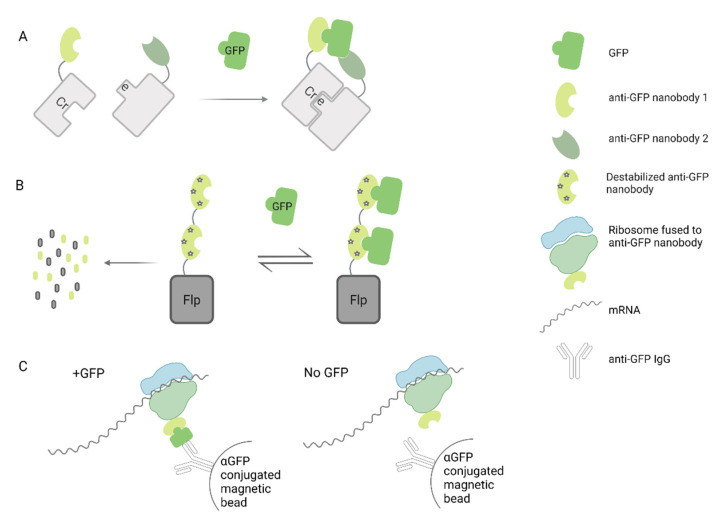
Schematic representation of research approaches using GFP/nanobodies scaffolds delivered by AAV vectors. (**A**) Cre recombinase dependent on GFP system, (**B**) Flp recombinase dependent on GFP system, (**C**) Immunoprecipitation of ribosome complexes dependent on GFP [82] and [85].

A more complex approach combining nanobodies and fluorescent proteins has been developed by Tang et al. to generate scaffolds for cell specific gene manipulation [133]. In this case, they generated green fluorescent protein (GFP)-specific nanobodies fused to complementing domains of transcription factors. Co-expression of these modified nanobodies and GFP in the cells allowed the formation of active transcription complexes that could also be easily visualized. This strategy has also been applied to control the activity of Cre recombinase by using two fragments of this protein, each one fused to a different GFP-specific nanobody [84]. In this system, called CRE-DOG, binding of each modified nanobody to GFP would allow the assembly of a functional Cre recombinase, leading to Cre/lox recombination (Figure 3A). As a proof of concept, they used an AAV vector to deliver CRE-DOG into the retina of transgenic mice expressing GFP in retinal ganglion cells, along with a second AAV expressing a Cre responsive element that would produce red fluorescence after recombination. As expected, recombination was specifically observed in GFP-expressing cells. The authors also proved that their results could be extrapolated to diverse brain regions, demonstrating that this could be a versatile strategy to manipulate genes in other GFP transgenic mice available for research.

The same group has also created a conditional system in which the stability of a nanobody depends upon binding an antigen of interest [83]. For that purpose, they identified framework mutations that caused destabilization of nanobodies in the absence of the target antigen, leading to degradation by the ubiquitin proteasome system. Interestingly, any protein fused to the destabilized nanobody will also be degraded. This strategy was applied to control the stability of Flp recombinase by its fusion to a dimer of destabilized anti-GFP nanobodies. Therefore, Fpl-mediated recombination was only possible in GFP-expressing cells (Figure 3B). By using this unique technology delivered by AAV vectors, they were able to express exogenous genes into GFP-expressing cells in the brain of transgenic mice.

Finally, Ekstrand et al. showed that transgenic mice expressing an anti-GFP nanobody fused to the large ribosomal subunit in a neuro-specific way, allows for gene expression profiling of GFP-expressing neurons, by capturing GFP complexes (including ribosomes and translating mRNAs) through immunoprecipitation (Figure 3C) [85]. The injection of a retrogradely transported adenovirus vector expressing GFP into a specific area of the brain allowed them to selectively profile pre-synaptic neurons projecting to that area. Going further, they were able to specifically determine the gene expression profile of dopaminergic neurons by using a Cre-conditional AAV5 vector to deliver the ribosome–nanobody construct into the brain of transgenic mice that express Cre recombinase under the control of the dopamine transporter promoter [85]. This work opens up the prospect of translational profiling of other neuronal subpopulations based on their connectivity.

## 9. Using Nanobodies to Re-Target AAV Vectors

An ideal in vivo gene therapy strategy involves not only an efficient expression of the therapeutic transgene, but also the specificity of such expression in the cells or tissue of choice. The tropism of AAVs is ubiquitous for most serotypes, and can be effectively exploited in some cases, such as when seeking high transgene expression in muscle by administering AAV1 or AAV8 vectors intramuscularly [7]. However, there are a wide variety of diseases in which the target cells are poorly transduced in vivo with the available serotypes, or in which administration of AAV intravenously is necessary, resulting in the transduction of unwanted tissues as a side effect. In these cases, the modification of the AAV capsid is proposed, in order to eliminate its natural tropism and provide it with a new and specific one. By means of genetic engineering, and with the help of the 3D structures of the different capsid serotypes, it is possible to insert peptides [134] or protein domains in the GH2/GH3 loop of VP1, the most prominent protrusion of the viral capsid [135].

To achieve a highly specific tropism, the recombinant fusion of antibodies to the aforementioned loop is a very attractive alternative. Unfortunately, this is a difficult task when using conventional antibodies, since their variable domain is made up of two different polypeptide chains and they are too large [136]. However, nanobodies have been proven to be good candidates to be fused with AAV2-VP1 [86].

Eichhoff et al. [86] retargeted the natural tropism of AAV2 capsid by inserting specific nanobodies for three structurally different membrane proteins: a GPI-anchored ectoenzyme (ARTC2.2), a single-span ectoenzyme type II (CD38), and a homotrimeric multispan ion channel (P2X7). To this end, they genetically replaced seven amino acids of the VP1-GH2/GH3 loop (amino acids 453–459) by residues 110–130 of the corresponding nanobody. To prevent binding to the AAV2 natural receptor (heparan sulfate proteoglycan), arginines 585 and 588 were replaced by alanine. They showed that engineered AAVs were capable of transducing stably transfected HEK293 cells expressing the respective target proteins, achieving a 10–500-fold increase in transduction efficiency compared to the parental serotype. Similar results were obtained using other cells that express these proteins, such as mouse mammary carcinoma and lymphoma cell lines, as well as primary bone marrow cells that endogenously express CD38 or P2X7. The authors demonstrated that they could not only direct AAV tropism towards new cells, but also that retargeting was highly specific for cells expressing the corresponding protein. Interestingly, the nanobody-VP1 (AAV2) fusion protein was effectively incorporated into the capsid of other serotypes (AAV8, AAV9, or AAV1P5), resulting in high yields of the corresponding mosaic AAV viral particles and leading to specific retargeting in vitro. Although these are promising results, in vivo studies have yet to be performed.

## 10. Conclusions and Future Perspectives

In modern medicine, a combination of new technologies usually allows the rapid advancement of efficient therapeutic treatments. One recent example is the combination of technologies to produce highly stable mRNA molecules and potent lipid formulations for in vivo delivery of nucleic acids, which has led to the development of efficient COVID-19 mRNA-based vaccines. In this review, we have shown that the numerous advantageous properties of nanobodies can be enhanced when they are delivered in vivo by AAV vectors able to: i) provide very long-term expression, and ii) deliver nanobodies both intra- or extra-cellularly. In this way, AAV nanobody delivery has shown great potential to cure a plethora of pathologies in preclinical models, ranging from genetic diseases to prevalent acquired malignancies, such as cancer, cardiovascular, infectious, and neurodegenerative diseases. In all these cases, the property of nanobodies to “block” a specific ligand, being it a foreign viral protein or an endogenous enzyme or protein with deleterious activity, is used to mediate a therapeutic effect that is maintained in time thanks to AAV delivery. In many of these cases, the use of nanobody dimers or multimers has shown more potency over the use of monomers, suggesting that future treatments based on this technology will probably require this strategy. In some cases, nanobodies have also been fused to proteins that increase their stability and half-life, something particularly interesting given their rapid renal elimination when delivered systemically. The good news is that, with nanobodies being so small, AAV vectors can easily accommodate genes coding for several nanobodies or for nanobodies fused to other proteins. The use of AAV-delivered nanobodies is not only limited to therapeutic purposes but has also found a niche in more basic research. This is due to the fact that nanobodies can also tamper with the activity of normal cellular proteins in a very specific way, which facilitates the discovery of new biological functions. A particular application of nanobodies for research is the use of protein domains fused to nanobodies specific for reporter proteins, such as GFP. In this case, the functional protein can only be reconstituted when their nanobody-fused domains bind to GFP, allowing to study more precisely its function or limiting its activity in reporter cells.

Although AAV delivery of nanobodies is still limited to a few preclinical models, the promising results obtained in these studies, together with the high safety profile shown by AAV vectors and nanobodies in clinical trials, suggest that this type of new therapy could soon be translated to the clinic.

## Figures and Tables

**Table 1:** Therapeutic nanobodies in clinical trials.

Drug Name(s)	Format	Nanobody’s Target(s)	Indication	Current Status	Clinical Trial	Sponsor/Ref.
Caplacizumab ALX-0081 ALX-0681	Bivalent, monospecific	Von Willebrand factor-A	Acquired thrombotic thrombocytopenia purpura	FDA/EMA approved	NCT02553317 NCT02878603	Ablynx [54]
KN046	Tetravalent, bispecific, Fc-fusion protein	CTLA-4, PD-L1	Advanced solid tumors and lymphoma	Phase II/III	NCT03872791 NCT04474119 NCT04925947	Alphamab, Weill Medical College [55]
Ozoralizumab ATN-103	Trivalent, bispecific	TNFα (2), HSA	Rheumatoid arthritis	Phase II	NCT00959036 NCT01007175	Ablynx
Vobarilizumab ALX-0061	Bivalent, bispecific	IL-6R, HSA	Rheumatoid arthritis, systemic lupus erythematosus	Phase II	NCT02287922 NCT02437890	Ablynx [56]
Sonelokimab M1095	Trivalent, bispecific	IL-17F, IL-17A/F, HSA	Psoriasis	Phase II	NCT02156466 NCT03384745	Merck KGaA, Bond Avillion 2 Development LP [57]
Lulizumab BMS931699	Monomeric, pegylated	CD28	Systemic lupus erythematosus, kidney trasplantation	Phase II	NCT02265744 NCT04903054	Bristol-Myers Squibb [58]
ALX-0171	Trivalent, monospecific	RSV F-protein	RSV lower respiratory tract infection	Phase II	NCT02309320 NCT02979431	Ablynx [59]
LMN-101	Monomeric	FLaA flagellin of *Campylobacter jejuni*	*C. jejuni* infection	Phase II	NCT04182490	Lumen Bioscience
ARP1 VHH batch 203027	Monomeric	Rotavirus	Rotavirus infection	Phase II	NCT01259765	Int. Centre for Diarrhoeal Disease Research-Bangladesh [60]
Envolimab KN035	Monospecific, Fc-fusion protein	PD-L1	Advanced solid tumors, multiple primary neoplasm	Phase II	NCT03667170 NCT04182789 NCT04891198	Alphamab, 3D Medicines [61]
INBRX-109	Tetravalent, monospecific, Fc-fusion protein	Death receptor 5	Advanced solid tumors, conventional chondrosarcoma	Phase I/II	NCT03715933 NCT04950075	Inhibrx
KN044	Monospecific, Fc-fusion protein	CTLA-4	Advanced solid tumors	Phase I	NCT04126590	Intellicrown Pharm.
ALX-0141	Trivalent, bispecific	RANKL (2), HSA	Osteoporosis and bone metastasis	Phase I		Ablynx
M6495	Bivalent, bispecific	ADAMTS-5, HSA	Osteoarthritis	Phase I	NCT03583346	Merck KGaA [62]
ES101 INBRX-105	Tetravalent, bispecific, Fc-fusion protein	PD-L1, CD137	Advanced solid tumors	Phase I	NCT03809624 NCT04009460	Elpiscience, Inhibrx
ES102 INBRX-106	Hexavalent, monospecific, Fc-fusion protein	OX40	Advanced solid tumors	Phase I	NCT04198766 NCT04730843	Elpiscience, Inhibrx
BCMA nanobody CAR-T cells	Nanobody-based chimeric antigen receptor	BCMA	Relapsed/Refractory Myeloma	Phase I	NCT03664661	Henan Cancer Hospital
CD19/CD20 bispecific CAR-T cells	Nanobody-based bispecific chimeric antigen receptor	CD19/CD20	B-Cell Lymphoma	Phase I	NCT03881761	Henan Cancer Hospital
αPD1-MSLN-CAR T cells	MSLN-CAR T cells secreting anti-PD-1 nanobody	PD-1	Advanced solid tumors	Phase I	NCT04503980 NCT04489862	Shanghai Cell Therapy Group, Wuhan Union Hospital
ALX-0651	Biparatopic, monospecific	CXCR4	Multiple myeloma, non-Hodgkin’s lymphoma	Phase I terminated	NCT01374503	Ablynx
TAS266	Tetrameric	Death receptor 5	Advanced solid tumors	Phase I terminated	NCT01529307	Novartis Pharm. [51]

Abbreviations: CTLA-4, Cytotoxic T-Lymphocyte Antigen 4; PD-1, Programmed cell death 1; PD-L1, PD-1 ligand 1; TNF, tumor necrosis factor; HSA, human serum albumin; IL, interleukin; CD, cluster of differentiation; RSV, Respiratory Syncytial Virus; RANKL, ligand of receptor activator for nuclear factor-κ B; ADAMTS-5, a disintegrin and metalloproteinase with thrombospondin motifs-5; BCMA, B cell maturation antigen; MSLN, mesothelin; CXCR4, C-X-C motif chemokine receptor 4.

**Table 2:** AAVs to deliver nanobodies.

AAV ^a^	Nb Activity ^b^	Nb Conformation ^c^	Results ^d^	Ref.
9	Blocks gelsolin cleavage	Heterodimer	Improved muscle contraction in AGel mice	[72]
9	Blocks RyR2 phosphorylation	Monomer	Lower cardiac fibrosis after rat ischemic heart failure	[73]
5	Enhances α-syn degradation	Monomer-PEST	Motor function protection in PD rat model	[74]
8	Antithrombin blockade	Heterodimer	Restores hemostatic balance in hemophilia A/B mice	[75]
8	Neutralizes influenza virus HA	Homodimer	Protection against lethal influenza virus in mice	[76]
9		Multiple domains		[77]
8	Blocking of PD-1	Monomer	Protection against MC38 tumor challenge	[78]
1	P2X7 modulation	Dimer	Protection against EG7 tumor challenge	[79]
--	Blocks PTCH1 activity	Monomer	Hedgehog pathway activation	[80]
2/1	Actin targeting	Monomer-mNep	In vivo visualization of actin filaments	[81]
1	GFP targeting		Delivery of recombinases to GFP-expressing cells	[82]
2/1		Dimer-Flp	Provides Flp activity in GFP + cells	[83]
2/1		Monomer-Cre	Provides Cre activity in GFP + cells	[84]
5		Monomer-Rpl10a	Capture translating mRNAs from GFP + neurons	[85]
2/8/9	Nb fused to AAV capsid		Redirecting the specificity of AAV particles	[86]

^a^ AAV serotype; ^b^ The nanobody (Nb) target protein is underlined; RyR2, ryanodine receptor 2; α-syn, α-synuclein; HA, hemagglutinin; PD-1, programmed death protein-1; P2X7, ATP-gated ion channel P2X7; PTCH1, Hedgehog receptor Patched1; ^c^ PEST, PEST domain; mNep, far-red fluorescent protein mNeptune2; Rpl10a, large ribosomal subunit protein; ^d^ AGel, gelsolin amyloidosis; PD, Parkinson disease; GFP +, GFP-expressing cells.

## Data Availability

Not applicable.
